# Evaluation of the interventions on HIV case management and its association with cART adherence and disclosure of the disease status among HIV-positive adults under treatment

**DOI:** 10.1038/s41598-022-17905-6

**Published:** 2022-08-12

**Authors:** Awoke Seyoum Tegegne, Melkamu A. Zeru

**Affiliations:** Department of Statistics, Bahir Dar University, Bahir Dar, Ethiopia

**Keywords:** Biomarkers, Diseases, Health care, Health occupations, Medical research, Molecular medicine, Risk factors

## Abstract

The rate of prevalence of HIV among adults has been increasing in sub-Saharan African countries over the last decade. The objective of this study was to evaluate the interventions on HIV case management based on cART adherence and disclosure of HIV disease status among HIV-positive adults under treatment. A retrospective cohort longitudinal data was conducted on 792 randomly selected patients in the study area. Engagement of HIV-positive persons into care and achieving treatment outcomes such as the disclosure of HIV status and cART adherence were fundamental for HIV prevention strategy. The two response variables under the current investigation were evaluation of intervention on HIV case management interims cART adherence and disclosure of HIV status. Binary logistic regression was conducted for separate models. Among the predictors, age of patients (AOR = 1.020, 95% CI (1.016, 1.191); p value = 0.005), the number of follow-up (AOR = 1.014, 95% CI (1.023, 1.030); p value < 0.0001). CD4 cell count (AOR = 0.981; 95% CI (0.765, 0.971), p value < 0.01), Marital status (AOR = 1.013; 95% CI (1.002, 1.015), p value = 0.006), female patients (AOR = 1.014; 95% CI (1.001, 1.121), p value < 0.007), rural (AOR = 0.982; 95% CI (0.665, 0.998), p value = 0.004), non-educated adult patients (AOR = 0.950, 95% CI (0.92. 0.98). p value = 0.003), Non-existence of social violence (AOR = 1.012, 95% CI (1.008, 1.234), p value < 0.01), adult with non-opportunistic diseases (AOR = 1.021, 95% CI (1.002. 1.042). p value = 0.001) significantly affected the two response variables jointly. Interventions on HIV case management lead to an efficient continuum of successful treatment outcomes like disclosure of HIV status and cART adherence. Hence, HIV case management intervention and the two results had a positive association. HIV case management intervention should be given to younger patients, rural residents, and non-educated patients to disclose the disease status and to have a long life with the virus. Health-related education should be conducted for the community in general and for patients in particular on how HIV is transferred from an infected person to an uninfected one. This helps to reduce the stigma of patients and to deliver social support to patients.

## Introduction

HIV continues to be a serious global public health problem with 36.7 million people living with HIV, 1.8 million new infections, and 1 million people dying from HIV-related illnesses^1^. Among these, bout 19.4 million people are testified to live with HIV in Sub-Saharan Africa^2^.

The infection of HIV is the leading public health-related problem in Ethiopia. Amhara region, one of the eleven regions in the country, accounts for the highest number of people living with HIV. In the region, the overall incidence rate of new HIV infection is 6.9 per 1000 tested population^3^. Disclosing own HIV status is one of the indicators of outcomes of intervention in HIV case management and is crucial in reducing the transmission of the virus from infected to non-infected individuals. Disclosing the HIV status facilitates cART adherence to be effective^4^. Disclosure of the HIV status is one indicator of interventions on HIV case management and behavioral changes in adults and this further leads to being cART adherent^4^. Hence, the disclosure of the disease status facilitates the behavioral change in avoidance of fear of other individuals during taking pills and patients focus on the prescribed time for dietary and pills rather than considering who is present with them and these can be progressive due to the intervention of those concerned bodies^5^.

Self-disclosure of the HIV disease status generally has important effects on an individual's health, lowers stress, and leads to better psychological relief^5^. In the case of HIV/AIDS, individuals who disclose their HIV status are in better health conditions in terms of reproductive choices as well as psychosocial readiness^6^.

Successful treatment with positive outcomes requires a combination of interventions, and antiretroviral therapy together with effective care and support systems. HIV case management intervention strategies facilitate the patients to be cART adherent (food, time, and medication) which may improve the treatment outcomes also. Previous studies indicate that individuals who got support on HIV case management have better support for social, physical, and spiritual care is an important part of HIV/AIDS clinical management and this further leads the patients to disclose their disease status and better adherence to cART treatments^4^. Previously conducted studies indicate that disclosure may increase opportunities to receive social support, which may help individuals cope and recover from physical illness, decrease depressive symptoms due to HIV-related indications, and finally lead to being cART adherent^4,7^. Disclosure of HIV status to all societies living around them is also crucial for avoidance of HIV transmission and helps for good cART adherence^8^. Hence, the two outcomes of HIV case management intervention namely disclosure of HIV status and cART adherence are highly correlated and one complements the other.

Previous studies indicate that HIV-infected individuals who had close follow-ups on their prescribed health-related education disclosed their HIV status and for the patients to be free from mental depression and stress to take his/her medication on time without fear of other individuals living together^9^. This indicates that the intervention in HIV case management helped to facilitate the conditions for patients’ adherence to cART and disclosure of HIV status^10^. This is why the joint predictors of the two responses were initiated. A number of key issues may be raised in the study of adherence to cART and disclosure of HIV status determinants affecting jointly, and the development of interventions. Addressing these issues may provide valued information about which patients are most at risk for non-adherence and how adherence can be improved. It is well known that patients hidden their own disease status may not take treatment medication on time if he/she is with another individual at a time around them and this further leads to failure to medication adherence^11^.

Although the study area remains the home to most of the people under treatment (ART), there is limited attention in terms of support over and above antiretroviral treatment (ART) programs. Case management intervention is a special kind of support provided to HIV patients consists of the provision of support services such as how to get various laboratory tests performed, keeping up appointments, family issues including children and spousal, food, transportation, and establishing a special relationship between HIV service providers and their clients^12^. Many previous studies had used joint models for repeated outcomes of longitudinal responses and time to event^13^. Such studies did not investigate the evaluation of interventions in HIV case management based on the two longitudinal and correlated outcomes observed repeatedly from the same subject and lacked multivariate analysis of the two observed results. Joint modeling between two repeated measures has benefits in reducing type I error rates in numerous tests with repeated observation on the same subject and advances efficiency in approximating the unknown parameters^14^.

As far as the authors’ knowledge is concerned, no research has been conducted on the evaluation of interventions on HIV case management in terms of the two correlated longitudinal outcome variables, in the study area. Therefore, the current investigation was conducted with the objective of evaluating the interventions in HIV case management based on the performance of the two HIV case management outcomes, disclosure of HIV status, and adherence to cART (dietary, time, and medication). The result obtained in the current investigation helps to conduct special intervention strategies and is further important to strengthen the positive relationship between service providers and patients and to design interventional strategies.

## Materials and methods

### Study site and population

The study was conducted at Felege-Hiwot Teaching and Specialized Hospital located in North-western Ethiopia, Amhara Region. The hospital is a referral hospital in which many patients referred from district hospitals in the region. The hospital has regional laboratory where all HIV results in different district hospitals in the region are collected, processed, and organized to send to Ministry of health. There are about 6000 HIV infected adults treated at the hospital whose enrolments were between Sep and Jun/2012. Among these, about 2000 were under ART.

### Study design

A retrospective cohort study design was conducted to assess joint predictors of disclosure of HIV status and cART adherence among HIV infected adults enrolled in the first 10 months of 2012 and followed up to June 2017. Both separate and joint models were used in data analysis.

### Inclusion criteria

HIV patients aged 15 years and above, registered in the first 10 months of 2012 and initiated their cART in the hospital from September 2012 to June 2017 who had at least 2 follow-ups at Felege-Hiwot Referral and Specialized Hospital, were considered under this study.

### Sample size and sampling technique

Out of the targeted population, 792 were selected using a stratified random sampling technique considering their residence area as strata using a 95% level of confidence and 5% marginal error^15^.

### Data quality and analysis strategy

The quality of the data was controlled by data controllers from the ART section of the hospital. Training about the way how to follow up on the quality of data was given to data controllers by the Ministry of Health. A pilot test on the consistency research questions was conducted on 35 random samples and some modifications to the questionnaires were made on the final data collection sheet.

### Data collection tools and extraction procedures

Before the required data has been collected, there was a discussion with the health staff at ART section in the hospital about the variables included in this investigation. The required data was extracted from each participants chart using data extraction format. The format was developed by an author in consultation with health staff. Data analysis was conducted using Statistical System Analysis (SAS) software version9.2.

### Variables under investigation

#### Response variable

The longitudinal response variable under investigation was the assessment of the evaluation of interventions on HIV case management expressed in terms of disclosure of HIV status and adherence to cART. Both the two HIV case management results were binary in nature and measured as follows; if an individual disclosed the HIV status to families, friends, and relatives including their sexual partner, it is leveled as disclosed (yes), otherwise (no). On the other hand, if patients adhered to at least 95% of the prescribed cART (time, dietary, and medication), it is cART adherent (yes) otherwise, non-adherent (no).

#### Predictor variables

The explanatory variables for the two HIV case management result variables were age in years, sex (male, female), marital status (living with a partner, living without a partner), cell phone ownership (yes, no), weight in kilogram, baseline CD4 cell count in cells/mm^3^, disclosure status of the disease (yes, no), level of education (non-educated, educated), residence (rural, urban), clinical WHO stages (stage1, stage2, stage3, and stage4), cART adherence (adherent, non-adherent), income level (low, middle and high), follow up times/visits (1, 2…0.23), mental depression/stress (yes, no), social discrimination (yes, no), HIV case management intervention given to patients disclosed the disease (yes, no).

#### Impact of dropouts on the analysis

Patients who defaulted from cART treatment develop drug-resistant virus which ultimately leads to a bad response from the treatment and finally resulted to be death. Missing observations were tested using logistic regression to assess whether the missing values were independent of the past result.

#### Model selection

In model selection, all predictors were included in the model and fitted each product term obtained from predictors one at a time which helps to assess the interaction effect of covariates on the response variable.

Before conducting a joint model, separate models were done for each response using binary logistic regression models for each of the two responses^16^. The covariance structure and the magnitude of residual errors were also measured in model selection. In this regard, the model with the smallest individual residual inconsistency was selected^17^.

### Models used separate analyses of the two outcomes

In this investigation, an analysis of binary data in terms of the binomial distributions with logit transformation was conducted. The result is a binomial response conducted with a logistic regression model with a logit link function.

### Formulation of joint modeling

To construct a joint model of two outcome variables, let be the first response (disclosure of HIV status) and the second response (adherence to cART).

For bivariate response vector for the same subject i, let $${{\varvec{y}}}_{1ij}$$ = ($${y}_{1i1},{y}_{1i2},\dots .., {y}_{1{in}_{i}}{)}^{T}$$ and $${y}_{2ij}$$= ($${y}_{2i1},{y}_{2i2},\dots .., {y}_{2i{n}_{i}}{)}^{T}$$ be repeated measures for the two responses; where $${y}_{kij}$$= ($${y}_{1ij}^{T}, {y}_{2ij}^{T}{)}^{T}$$ (k = 1, 2).

Hence, we can have two possible alternatives for the formulation of a joint model namely the conditional argument approach and the direct formulation approach. In the conditional argument approach, the joint model can be formulated by factoring out the given distribution as marginal and conditional components with the introduction of the probit approach. In the direct formulation approach, the Placket–Dal approach (placket latent variable) can be considered for modelling bivariate responses^18^.

The joint generalized linear mixed effect models, assuming that each outcome and the univariate models are combined through the specification of joint multivariate distribution for all random effects.

For the assessment of the relation between two responses (disclosure of HIV status and adherence to cART), the joint GLMM model was fitted^19^. In this model, the association between the two outcomes was quantified through the random effect given that separate random intercept for each outcome variable has been conducted and merging them by imposing joint multivariate distribution on the random intercept^20^. The association between intervention on HIV case management and the two response variables was investigated to assess how the intervention on HIV case management was progressive in terms of disclosure of HIV status and cART adherence.

### Experiments conducted in current investigation

All experiments were performed in accordance with relevant guidelines and regulations.

### Ethical approval and informed consent to participate

Informed consent from participants was not obtained because of the use of secondary data collected by health staff for medical purpose. An investigator used a secondary data without names of patients to protect their privacy and confidentiality. Ethical approval certificate had been obtained from Bahir Dar University Ethical board, Bahir Dar, Ethiopia with reference number: RCS/1412/2012. Hence, the current study was approved by the board indicated above.

## Results

Among the sample of 792 patients: 40.9% were rural residents; 50.6% were females; 56.3% were living with their partners; 21% of the patients disclosed their disease status to family members, 49.2% were owners of cell phones, 25.5% were cART adherent, only 11.5% had high income and 20.6% had no education. Among the participants, 20.7% declared that there was social discrimination by societies living with them and about 46.2% said that there was no HIV case management intervention for those patients who disclosed their disease. Some of the participants (47.3%) declared that there was mental depression/stress because of the drug at the initial time of the cART. The average (median) weight was 58 kg (IQR (52, 64)), average years of all patients was 36 years (IQR (28, 48)). The average (median) baseline CD4 cell count for all patients was 134 cells/mm^3^ (IQR (113,180). The baseline characteristics of respondents are indicated in Table 1.

**Table 1:** Baseline socio-demographic, economic and clinical variables (n = 792).

Variables	Average	No (%)
Weight (kg)	58.1 (45–70)	–
Base line CD4 cells/mm^3^	148.7 (113–180)	–
Age (years)	74.3 (48–78)	–
Follow-up times	23 visits	–
First month/initial CD4 cell count change/mm^3^	16.6 (12–26)	–
**Sex**		
Male		392 (49.4)
Female		400 (50.6)
**Educational status**		
Non-educated		163 (20.6)
Educated		629 (79.4)
**Residence area**		
Urban		468 (59.1)
Rural		324 (40.9)
**Marital status**		
Living with partner		446 (56.3)
Living without Partner		346 (43.7)
**Existence of social discrimination**		
Yes		164(20.7)
No		628(79.3)
**Intervention on HIV case management**		
Yes		426(53.8)
No		366(46.2)
**Existence of mental depression/stress at initial time**		
Yes		375(47.3)
No		417(52.7)
**WHO HIV stages**		
Stage I		101 (12.8)
Stage II		259 (32.7)
Stage III		199 (25.1)
Stage IV		233 (29.4)
**Disclosure of HIV status**		
Yes		166 (21.0)
No		426 (79.0)
**Ownership of cell phone**		
Yes		390 (49.2)
No		402 (50.8)
**Adherence to cART**		
Adherent		202 (25.5)
Non-adherent		590 (74.5)

In the analysis, among the patients who disclosed the disease, 65% reported that they got better social support from communities around them. Similarly, the mental depression of participants was also invented using Beck’s depression inventory scale at each visit and 178 (22.5%) were mentally depressed.

The nature of the missingness pattern in the current investigation was tested using a logistic regression model and is known to be monotone (dropouts). The pattern indicates that there was no missing observation in the first two visits and the number of dropouts increased linearly as follow-up times/visits increased. The result in this regard revealed that dropouts were not affected by the previous outcomes (χ^2^_1_ = 0.3018, p = 0.762). Hence, the trend of missingness was Missed Completely at Random (MCAR).

Missing data were handled using multiple computation techniques. Parameter estimates for the two responses were inducted separately before the construction of joint models as shown in Table 2.

**Table 2:** Parameter estimates for marginal models of disclosure of HIV status and adherence to cART.

Variables	Disclosure of HIV status			Adherence to cART		
	Estimates	St. error	p value	Estimates	St. error	p value
Intercept	3.014	0.703	< 0.01*	0.922	0.245	< 0.01*
Age	0.022	0.611	0.003*	0.122	0.013	< 0.01*
Weight	− 0.023	0.814	0.001*	0.031	0.016	< 0.01*
Baseline CD4 cell count	0.011	1.413	< 0.001*	0.022	0.014	< 0.01*
Number of follow-ups	0.032	0.943	< 0.001*	0.033	1.038	< 0.01*
**Marital status (Ref. = without partner)**						
With partners	0.023	1.603	0.005*	− 0.012	0.051	0.021*
Sex (Ref. = male)						
Female	0.015	0.713	< 0.001	0.014	1.104	< 0.001*
**Existence of social discrimination (Ref. = yes)**						
No	0.046	1.435	< 0.01*	− 0.132	1.023	0.002*
**Intervention on HIV case management (Ref. = yes)**						
No	− 0.031	0.763	0.002*	− 0.031	0.231	0.031*
**Existence of mental depression/stress (Ref. = yes)**						
No	0.021	0.923	0.013*	0.021	0.231	0.003*
**Residence area (Ref. = Urban)**						
Rural	− 0.016	0.921	0.046*	− 0.135	1.432	0.148
**Level of education (Ref. = educated)**						
Non-educated	− 0.024	0.814	< 0.021*	− 0.125	0.156	0.512*
**Ownership of cell phone (Ref. = No)**						
Yes	− 0.035	1.906	< 0.018*	0.725	1.091	< 0.014*
**WHO stages (Ref. = stage IV)**						
Stage I	− 0.138	0.818	< 0.016*	− 0.194	1.013	0.072
Stage II	− 0.142	1.916	< 0.073	0.246	0.092	0.026
Stage III	− 0.19	1.715	< 0.059	0.158	0.096	0.094

*significant at 5% level of confidence.

The results in Table 2 show the separate or marginal models for the two variables of interest, considering binary logistic regression for both responses. The main effects, age, weight, baseline CD4 cell count, the number of follow-up visits, sex, marital status, existence of social discrimination, intervention in HIV case management, existence of stress, and cell phone ownership considerably influenced both outcomes.

To assess the joint determinants of the two responses, a joint multivariate distribution becomes more relevant. Developing models for the two response variables with uncorrelated random intercepts gives results for an initial parameter estimate. The results obtained by applying this procedure are indicated in Table 3. The analysis was conducted using log-likelihood functions with the Laplace approximation. The conditional independence of random-effects models conducted in this analysis shows that the GLMMs approach could be an extensive, separate and random effect. The SAS procedure that uses generalized mixed effect models allows the joint distribution to be constructed for the random effects from the two separate models^21^.

**Table 3:** Parameter estimates for conditional independence of random intercepts model with disclosure of HIV status data and adherence to cRRT data.

Parameter	Disclosure of HIV status			Adherence to cART		
	Estimates	St. error	p value	Estimates	St. error	p value
Intercept	3.014	0.003	< 0.01*	0.922	0.245	< 0.001*
Age	0.025	0.011	0.003*	0.122	0.013	< 0.001*
Weight	− 0.021	0.014	0.001*	0.031	0.016	0.001*
Baseline CD4 cell count	0.014	0.013	< 0.001*	0.022	0.014	< 0.001*
Number of follow-ups	0.032	0.043	< 0.001*	0.033	0.038	0.012*
**Marital status (Ref. = Without partner)**						
With partners	0.023	0.035	0.005*	− 0.012	0.051	0.021*
Sex (Ref. = Male						
Female	0.01	0.016	< 0.001	0.014	0.104	< 0.001
**Existence of social discrimination (Ref. = yes)**						
No	0.046	0.435	< 0.001	− 0.132	0.023	0.002*
**Intervention on HIV case management (Ref. = yes)**						
No	− 0.031	0.763	0.002	− 0.031	0.231	0.031*
**Existence of mental depression/stress (Ref. = yes)**						
No	0.021	0.923	0.013	0.021	0.231	0.003*
**Residence area (Ref. = Urban)**						
Rural	− 0.015	0.016	0.045*	0.134	0.013	0.014
**Level of education (Ref. = educated)**						
Non-educated	− 0.026	0.015	< 0.012*	− 0.125	0.154	0.012*
**Ownership of cell phone (Ref. = No)**						
Yes	− 0.034	0.204	< 0.013*	0.725	0.091	< 0.013*
**WHO stages (Ref. = stage IV)**						
Stage I	0.137	0.015	< 0.014*	− 0.192	0.013	0.072
Stage II	0.146	0.016	< 0.041	0.246	0.094	0.025
Stage III	0.104	0.014	< 0.015*	0.156	0.092	0.092

*Significant at 95% CI for both outcomes.

This shows the way how to construct multivariate longitudinal data by supposing separate random effects from a generalized mixed approach for each outcome variable. The formulation of a joint model by striking a joint multivariate distribution helps in developing a joint multivariate distribution in the random effects of the two separate models. Parameter estimates for the conditional independence of the random intercept model for disclosure of HIV status data and adherence to cART are indicated in Table 3.

As shown in Table 3, age of patients, weight, CD4 cell count at baseline, the number of follow-ups, cell phone ownership, the existence of social support, social discrimination, the existence of stress, and sex significantly affected both responses variables. The identical sign for the two predictors indicates that they are positively correlated to each other. Hence, a patient who disclosed the HIV status can be adherent to cART and an adherent to cART patient encourages to be disclosed the HIV status.

However, the results in Table 3 revealed that the conditional independence assumption was not flexible (it is restrictive), and considering the conditional dependence assumption provides a relaxed assumption and it gives an alternative approach by re-considering the joint random intercepts model with potentially associated errors.

Introducing the restricted dependence of one outcome in terms of the other using a linear predictor provides and formulates joint predictors of two longitudinal response variables with the possible error values created^21–23^. This approach is also helpful to evaluate the observed correlation between the two responses increasing from the association of the random intercepts. Hence, a generalized linear mixed model was fitted for disclosure of HIV status as a variable of interest including adherence to cART as a linear predictor. Table 4 indicates that disclosure of HIV status is positively correlated with adherence to cART (p value < 0.001). That is, if the status of HIV was disclosed, an individual who disclosed the disease can strictly adhere to the prescribed medication by the health staff and respected the due date, time, and dietary instruction without fear of anyone. On the other hand, if patients adhere to cART medication properly, then they would have a motivation to disclose their disease status to families, friends, relatives, or sex partners^24,25^.

**Table 4:** Parameter estimates for disclosure of HIV status data using a linear predictor.

Parameter	Estimates	St. error	Adjusted odds ratio (AOR)	Wald 95% CI		p value
Intercept	3.011	0.037	20.29	51.53	58.62	< 0.001*
Age	0.022	0.267	1.020	1.016	1.19	0.005*
Weight	− 0.026	0.865	1.024	0.015	1.01	0.082
Baseline CD4 cell count	− 0.023	0.764	0.981	0.765	0.97	< 0.001*
Follow-up times	0.014	0.517	1.014	1.023	1.03	< 0.001*
**Marital status (Ref. = without partner)**						
With partners	0.011	0.715	1.013	1.002	1.015	0.006
**Sex (Ref. = male)**						
Female	0.014	0.453	1.014	0.015	1.02	0.007
**Existence of social discrimination (Ref. = yes)**						
No	0.046	0.435	1.041	1.001	1.121	< 0.001*
**Intervention for patients disclosed disease (Ref. = yes)**						
No	− 0.034	0.546	0.970	0.765	0.998	< 0.001*
**Existence of mental depression/stress(Ref. = yes)**						
No	0.011	0.462	1.010	1.001	1.131	0.001*
**Opportunistic infectious disease (Ref. Yes)**						
No	0.021	0.082	1.021	1.002	1.042	0.001*
**Residence area (Ref. = urban)**						
Rural	− 0.021	0.011	0.982	0.965	0.998	0.004*
**Level of education (Ref. = tertiary education)**						
Non-educated	− 0.052	0.012	0.951	0.924	0.987	0.003*
**Level of income(Ref. = high income)**						
Middle income	− 0.01	0.013	0.991	0.015	1.012	0.006
Low income	− 0.014	0.006	0.992	0.986	1.102	0.103
**Ownership of cell phone (Ref. = yes)**						
No	− 0.024	0.307	0.982	0.525	0.997	< 0.001*
**Adherence to cART (Ref. = non-adherent)**						
Adherent	0.063	0.514	1.065	1.061	1.073	< 0.001*
**WHO stages (Ref. = stage IV)**						
Stage I	0.124	0.013	1.136	1.123	1.142	0.062
Stage II	0.123	0.012	1.132	1.124	1.052	0.061
Stage III	0.102	0.043	1.118	0.092	1.103	0.093

*Significant at 95% CI for both outcomes, ${e}^{\beta }$ = AOR.

Finally, the parameter estimates of disclosure of HIV status considering adherence to cART as a linear predictor are indicated in Table 4. Table 4 indicates that predictors like age of patients, baseline CD4 cell count, the number of followed-up visits, marital status, sex, residence area, cell phone ownership, intervention in HIV case management, social discrimination, mental stress, and level of adherence to HAART had a significant effect on the variable of interest.

As the age of patients increased by 1 year, the expected odds of being disclosed the status was increased by 2% assuming that the other things remain constant (AOR = 1.020, 95% CI (1.016,1.191); p value = 0.005). Similarly, as the number of follow-up visits increased by one unit, the expected value of the odds of being disclosed the disease was increased by 1.4% provided that patients adhered properly to the prescribed medication by health staff (AOR = 1.014, 95% CI (1.023, 1.030); p value < 0.01). The level of being adherent was not constant and it was dynamic, hence as visiting time of a patient increased by one unit, the expected odds of being adherence for patients increased by 6.5%, given that the other covariates remains constant(AOR = 1.065, 95% CI (1.061, 1.073) and p value < 0.01).

As baseline CD4 cell count increased by one unit, the expected value of odds of being exposed the HIV status was decreased by 1.9% given the other covariates constant (AOR = 0.981; 95% CI (0.765, 0.971), p value < 0.01).

Marital status had a significant effect on the variable of interest. Hence, comparing patients living with their partners and without partners, patients living with their partners have a high possibility of disclosing the disease to families, friends, and relatives including sexual partners. The expected possibility of odds of disclosing the HIV status by patients living with partners was increased by 1,3% as compared to patients living without partners(AOR = 1.013; 95% CI (1.002, 1.015), p value = 0.006).

Comparing female HIV-infected patients with males, the expected number of disclosed HIV status by females was increased by 1.4% to males given the other covariates constant(AOR = 1.014; 95% CI (1.001, 1.121), p value < 0.007). however, the expected odds of being disclosed HIV status by rural patients was decreased by 1.8% as compared to urban patients, given the other covariates constant(AOR = 0.982; 95% CI (0.665, 0.998), p value = 0.004).

The expected odds of being disclosed the HIV status by non-educated adult patients was decreased by 5% as compared to educated adults, keeping the other things constant(AOR = 0.950, 95% CI (0.92. 0.98). p value = 0.003).

Similarly, The expected odds of being disclosed the HIV status by cART non-adherent adult patients was decreased by 6% as compared to cART adherent adults, keeping the other things constant(AOR = 0.940, 95% CI (0.61. 0.97). p value < 0.001).

The existence of social violence had a statistically significant effect on HIV-positive adults not disclosed their status of HIV disease for sexual partners. Hence, the expected odds of being disclosed the HIV status for sexual partners by HIV-infected individuals, where there is no social violence, was increased by 1.2% as compared to those HIV infected adults living in societies, where there is social violence, keeping the other things constant (AOR = 1.012, 95% CI (1.008, 1.234), p value < 0.01).

In the current investigation, intervention in HIV case management had a significant effect on both the two response variables. Hence, comparing those patients who got special intervention/support by health staff, communities, families, and sexual partners with those who did not get such intervention, the expected odds of being disclosed by patients who did not get intervention in HIV case management was decreased by 3% as compared to those patients who got special interventions (AOR = 0.970, 95% CI (0.765, 0.998) and p value < 0.01).

The expected odds of being disclosed the HIV status by non-opportunistic diseases adult patients was increased by 2.1% as compared to opportunistic infectious disease adults, keeping the other things constant(AOR = 1.021, 95% CI (1.002. 1.042). p value = 0.001).

WHO stages had also a statistically significant effect on the disclosure of the level of HIV status for sexual partners. Hence, the expected odds of being disclosed the HIV status by adult patients whose WHO stage 1 was decreased by 11.3% as compared to WHO stage 4 keeping the other variables constant. Similarly, the expected odds of being disclosed the HIV status by adult patients whose WHO stage 2 was decreased by 12.2% as compared to WHO stage 4 keeping the other variables constant, and the expected odds of being disclosed the HIV status by adult patients whose WHO stage 3 was decreased by 9.5% as compared to WHO stage 4 keeping the other variables constant.

## Discussion

The current study tried to evaluate the intervention on HIV case management expressed interims of disclosure of HIV status and adherence to cART for HIV-positive adults under treatment. The extent of disclosure of their HIV status indicates that 79% of them did not disclose their HIV status to people around them. Similarly, the level of adherence to cART indicates that only 25.5% of the patients are adherent to cART. The level of intervention on HIV case management was 53% and needs special strategies for patients to be cART adherent and to disclose their disease status. Significance predictors for disclosure of the HIV status and cART adherence have been discussed below.

Age significantly affects the level of disclosure of the HIV-positive status for people living with HIV. As age increases, the disclosure levels of the disease status also increase. It is known that sexual intercourse decrease as the age of an individual increase and this may encourage disclosing the disease to people living around them. Hence, being older, HIV-infected individuals are more likely to have a steady sexual partner, and this contributes to an increase in the rate of disclosure^26,27^. Older HIV-positive patients with a high probability of disclosing the disease encouraged patients to be more adherent without any fears of others living together. Another previously conducted research indicates that the younger age group may not go for HIV testing and such people may not disclose their status unknowingly^28^. This further indicates that intervention in HIV case management becomes effective for aged HIV patients.

HIV-positive people with a high number of CD4 cell counts feel comfortable and healthy as compared to those with a low number of CD4 cell counts such people considered themselves HIV negative and they need not accept the diagnosis result given by the health staff. Hence, they are not volunteering to disclose their HIV status. The non-disclosure status of HIV-positive adults with high CD4 cell count leads to its less probable of being adherent to cART. The result in this regard is consistent with another previously conducted investigation^29^.

As visiting time of the heath institution increase, HIV-positive adults are encouraged to disclose their disease status because of their awareness and health-related education they got during every visiting time at health institutions (interventions). This result is similar to another previous research^30^. When HIV-positive adults visit the health institution as prescribed by the health staff, such people might be exposed to other individuals during visiting, and communication with such people encourages them to disclose the disease^31^. Visiting times have also a positive effect on patients being adherent to cART because of education and counseling given to patients at each visiting time. This indicates that cART adherence is dynamic which is progressive over time. In other words, as patients have more visiting time (follow-ups), they can get special intervention from the health staff and they may be encouraged to disclose their disease status to get combined intervention/support from the communities and families living together.

Marital status also significantly affects the degree of disclosure of HIV status. HIV-positive adults living with partners increase their willingness to disclose the status of their disease as compared to adults living without partners. The potential reason for this might be the fact that adults living with their partners feel more concerned about the health care of their partners. Disclosure of the disease for adults living with partners might help each other as a reminder to cART adherent and also important to remind the date when the partner should visit the health institution. The result in this regard is similar to previously conducted research^32^ and contradicted another research^33^. Hence, this result needs further investigation. Disclosure of HIV status empowers couples to make knowledgeable reproductive health varieties that may ultimately lower the number of unplanned pregnancies among HIV-positive couples, and even reduce the risk of HIV transmission from mother to child^34^.

Female HIV-positive adults are more likely to disclose their disease status to their partners as compared to males. The possible reason for this might be the fact that males need multiple partners as compared to females. Such needs discourage males to disclose their disease status and they need to hide the disease. Another reason may be the fact that females are willing to disclose their HIV status due to the responsibility of their concern for their partners’ health or to avoid their guilt^34^. The experience gained from pills taken for family planning contributed to females being cART adherent. This result is supported by previous research^35^ and contradicted by another investigation^35^. The reason for the contradicted result is that females hide their disease because of their fairness of stigma and discrimination^36^. This result also needs further investigation.

Urban HIV-positive adults are more likely to disclose the disease status as compared to rural HIV-positive adults. Urban patients might have a better understanding of disclosing the disease to get social support from the government and communities around them^36^. The culture in rural areas is more strict as compared to urban and the HIV-positive adults who disclosed their disease status might be discriminated against by society because of the reason that societies in the rural areas lack information on how and when the disease is transmitted from one individual to another^36^.

Social violence has a negative significant effect on HIV people not disclosing their disease status to their sexual partners. The potential reason for this might be that HIV-infected adults fear the trend that those individuals disclosed the disease violated by people living together^6^. Social violence also contributed a negative effect on HIV patients to be non-adherent (patients may not take pills whenever individuals are there). This indicates that disclosing the disease status may have its own negative effect (stigma) for the patients being discriminated against by the societies living together, where no awareness is created in the societies on how one can support patients who disclosed the disease.

Education plays a significant role in the variation of disclosure level of HIV status. Educated people are more likely to disclose the disease to people around them^19^. The potential reason for this might be the fact that such people have more information about the use of disclosing the disease to society, especially to their sexual partners^36^. Knowledge on how to prevent HIV transmission and how to get special intervention/support is important to disclose the HIV status and this further encourages the patients to be adherent to CART^36^.

## Conclusion

The majority of the participants (79%) under investigation did not disclose the HIV status which is a creditable strategy that will target those not likely to disclose will have to be evolved. Considering adherence to cART, only 25.5% were adherent to cART and about 53% had got intervention on HIV case management. Hence, the two low-performance outcomes require special intervention strategies on HIV case management and to identify, joint predictors of the two responses. HIV-positive patients under treatment who received case management support service have improved cART adherence and disclosure of disease status.

The association between the two responses in current investigation indicates that the two responses are highly correlated to each other. Patients who decided to disclose the disease are committed to being adherent to cART without fear of anyone living with them and such people are ready to get special intervention.

As recommendation, HIV case management intervention should be given to patients not disclosed the disease status and to non-adherent to cART HIV positive adults. Hence, health-related education for HIV-positive adults is crucial to disclose the HIV status and to be adherent to cART in order to have a long life with the virus. Special intervention related to knowledge on HIV transmission is also important to reduce the violence and discrimination of HIV-positive adults. The cART adherence is not a static but a dynamic which could increase or decrease over time according to some event life. The authors also recommend for further investigation why this could be happened. Intervention on HIV case management in ART regimen should be a priority in the study area.

This research was not without limitation, the data were taken in one treatment site, including the other treatment sites may provide additional information about the prevalence and predictors associated with why HIV infected individuals not disclose their HIV status to sexual partners, friends, relatives, families and generally to the society.

## Data Availability

The datasets used and/or analyzed during the current study are available from the corresponding author on reasonable request. The authors can attach the data upon request for result checkup purpose.
